# Characterization of the complete chloroplast genome of *Carallia diplopetala* (Rhizophoraceae)

**DOI:** 10.1080/23802359.2022.2135398

**Published:** 2022-10-27

**Authors:** Renjie Wang, Nanyan Liao, Xiongsheng Liu, Yi Qin, Yufei Xiao, Yong Wang, Ronglin Huang

**Affiliations:** a Guangxi Zhuang Autonomous Region Forestry Research Institute, Nanning, China; b Guangxi Fangcheng Golden, Camellias National Nature Reserve, Fangchenggang, China

**Keywords:** *Carallia diplopetala*, Rhizophoraceae, chloroplast genome, phylogeny

## Abstract

*Carallia diplopetala* (Rhizophoraceae) is an important economic tree species narrowly distributed endemic to East Asia. In this study, We generate the complete chloroplast genome of *C. diplopetala* using next-generation sequencing technology, which is 162,052 bp in size and consists of a large single copy (LSC) of 89,556 bp and a small single copy (SSC) of 18,814 bp, separated a pair of inverted repeats (IRb and IRa) of 26,841 bp. The overall GC content is 36.4%. A total of 130 genes are annotated, including 83 protein-coding genes, 37 tRNAs, eight rRNAs and two pseudogenes (ψ*ycf*1 and ψ*rps*19). The phylogenetic analysis indicated that *C. diplopetala* and *C. brachiate* formed a monophyletic clade with strong support and then sister to *Pellacalyx yunnanensis*. The plastome of *C. diplopetala* will provide informative genomic resources for further conservation applications.

*Carallia diplopetala* Hand.-Mazz. 1931 belongs to the mangrove family (Rhizophoraceae) and is an endemic species in East Asia with narrow distribution in China and Vietnam (Qin and Boufford 2007). This species provides a higher quality timber for furniture and industrial purposes (Liang et al. 2010). Its roots and leaves have been consumed as herbs in traditional Chinese medicine, and they are effective in treating rheumatism, bleeding and fever (Xiao and Wang 1992). With long-term over-excavation of the wild resources, the habitat has been severely fragmented and the populations have decreased significantly. Therefore, it was listed as an endangered species in China Species Red List (Fu 1992), requiring urgent conservation and restoration. However, there’s limited genomic information in regard to *C. diplopetala*. Here, we sequenced the complete chloroplast (cp) genome sequence of *C. diplopetala*, which will provide vital genetic information for sustainable management and utilization of this species.

The fresh leaves of *C. diplopetala* were collected in Guangxi Fangcheng Golden Camellias National Nature Reserve, Guangxi Province, China (21°45'37"N, 108°5′ 27"E). These samples collection was approved by the local management department. Total genomic DNA was extracted from silica-dried leaves by modified CTAB (hexadecyltrimethylammonium bromide) method (Doyle and Doyle 1987). A specimen was deposited at Guangxi Forestry Research Institute (contact: Ronglin Wang, e-mail: hrl3299@163.com, http://www.gxlky.com.cn/) under the voucher number hrl20210906001. The isolated genomic DNA was subsequently fragmented in length of 350 bp and sequenced on an Illumina Hiseq X-ten platform (San Diego, USA) at Novogene (Beijing, China). A total of 2 Gb raw Paired-end reads were yielded and *de novo* assembled into chloroplast genome using NOVOPlasty 4.3.1 (Dierckxsens et al. 2017). The genome annotation was performed by GeSeq (https://chlorobox.mpimp-golm.mpg.de/geseq.html; Tillich et al. 2017) and adjusted by manual in Geneious 11.1.5 (Kearse et al. 2012). The complete sequence data was submitted to National Center for Biotechnology Information (NCBI) under the accession number OM001094.

The chloroplast genome of *C. diplopetala* exhibited a typical double-stands circular structure with 162,052 bp in size, consisting of two duplicate inverted repeats (IRb and IRa) of 26,841 bp, isolated by the large and small single copy (LSC and SSC) regions of 89,556 bp and 18,814 bp, respectively. The overall GC content of plastome was 35.8%, with the GC contents of LSC, SSC and IRs at 33.2%, 29.6%, and 42.4%, respectively. A total of 130 genes were annotated, including 83 protein-coding genes, 37 transfer RNA genes (tRNA), eight ribosomal RNA genes (rRNA) and two pseudogenes (ψ*ycf*1 and ψ*rps*19). Among them, six protein-coding genes (*rpl*2, *rpl*23, *ycf*2, *ndh*B, *rps*7 and *rps*12), seven tRNA (*trnI-GAU*, *trnA-UGC*, *trnL-CAA*, *trnR-ACG*, *trnV-GAC*, *trnN-GUU* and *trnM-CAU)*, and four rRNA (*4.5S*, *5S*, *16S* and *23S* rRNA) were duplicated in the IR regions.

To reveal the phylogenetic relationship of *C. diplopetala* within Rhizophoraceae, we downloaded additional 17 species of Rhizophoraceae from Genbank, two species from Ctenolophonaceae (*Ctenolophon englerianus*, NC_049158) and Erythroxylaceae (*Erythroxylum novogranatens*, NC_030601) were chosen as outgroups according to the result of Xi et al. (2012) and Ruang-areerate et al. (2022). All sequences were aligned by MAFFT 7.409 (Katoh and Standley 2013). The phylogenetic analysis was conducted under the GTR + GAMMA model in RAxML with 1000 bootstrap replicates (Stamatakis 2014). Maximum likelihood (ML) phylogenetic tree indicated that *C. diplopetala* and *C. brachiate* formed a monophyletic clade with strong support and then sister to *Pellacalyx yunnanensis* (Figure 1). The topology of Rhizophoraceae is largely congruent with the result indicating from transcriptomes data (Guo et al. 2017).

**Figure 1.**
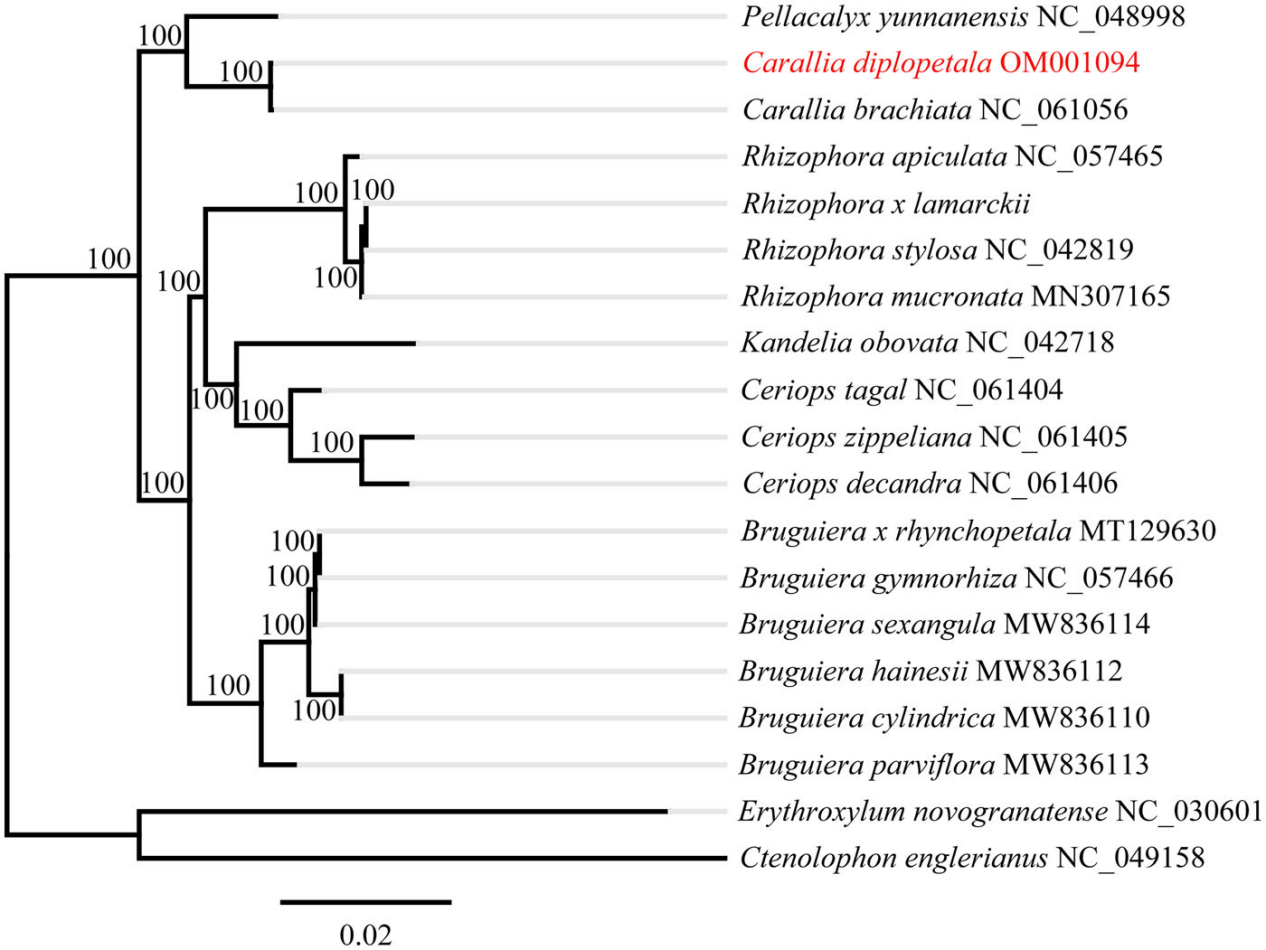
Phylogenetic tree of *Carallia diplopetala* using maximum likelihood method. Numbers on each node indicated the bootstrap support values after 1000 replicates. The following sequences were used: *Ceriops tagal* NC_061404, *Ceriops zippeliana* NC_061405, *Ceriops decandra* NC_061406 (Ruang-areerate et al. 2022); *Pellacalyx yunnanensis* NC_048998 (Zhang et al, 2019); *Kandelia obovata* NC_042718 (Chen et al. 2019); *Rhizophora stylosa* NC_042819 (Li et al. 2019); *Rhizophora x lamarckii* NC_046517, *Rhizophora stylosa* NC_042819, *Rhizophora mucronate* MN307165, *Rhizophora apiculate* NC_057465, *Bruguiera cylindrica* MW836110, *Bruguiera hainesii* MW836112, *Bruguiera parviflora* MW836113, *Bruguiera sexangular* MW836114, *Bruguiera gymnorhiza* NC_057466, *Carallia brachiate* NC_061056, *Carallia diplopetala* OM001094 (this study), *Erythroxylum novogranatense* NC_030601 and *Ctenolophon englerianus* NC_049158 (Wang et al. 2019).

## Data Availability

The genome sequence data that support the findings of this study are openly available in GenBank of NCBI at (https://www.ncbi.nlm.nih.gov/) under the accession number OM001094. The associated BioProject, SRA, and Bio-Sample numbers are PRJNA792453, SRR17332993 and SAMN24425643, respectively.
